# Growth hormone deficiency with advanced bone age: phenotypic interaction between *GHRH* receptor and *CYP21A2* mutations diagnosed by sanger and whole exome sequencing

**DOI:** 10.1590/2359-3997000000311

**Published:** 2017-12-01

**Authors:** Fernanda A. Correa, Marcela M. França, Qing Fang, Qianyi Ma, Tania A. Bachega, Andresa Rodrigues, Bilge A. Ozel, Jun Z. Li, Berenice B. Mendonca, Alexander A. L. Jorge, Luciani R. Carvalho, Sally A. Camper, Ivo J. P Arnhold

**Affiliations:** 1 Universidade de São Paulo Faculdade de Medicina Hospital das Clínicas São Paulo SP Brasil Unidade de Endocrinologia do Desenvolvimento, Laboratório de Hormônios e Genética Molecular LIM42, Disciplina de Endocrinologia, Hospital das Clínicas da Faculdade de Medicina da Universidade de São Paulo (HCFMUSP), São Paulo, SP Brasil; 2 University of Michigan Medical School Department of Human Genetics Ann Arbor MI USA Department of Human Genetics, University of Michigan Medical School, Ann Arbor, MI, USA; 3 Universidade de São Paulo Faculdade de Medicina Hospital das Clínicas São Paulo SP Brasil Unidade de Endocrinologia Genética, Laboratório de Endocrinologia Celular e Molecular LIM25, Disciplina de Endocrinologia, Hospital das Clínicas da Faculdade de Medicina da Universidade de São Paulo (HCFMUSP), São Paulo, SP Brasil

## Abstract

Isolated growth hormone deficiency (IGHD) is the most common pituitary hormone deficiency and, clinically, patients have delayed bone age. High sequence similarity between *CYP21A2* gene and *CYP21A1P* pseudogene poses difficulties for exome sequencing interpretation. A 7.5 year-old boy born to second-degree cousins presented with severe short stature (height SDS −3.7) and bone age of 6 years. Clonidine and combined pituitary stimulation tests revealed GH deficiency. Pituitary MRI was normal. The patient was successfully treated with rGH. Surprisingly, at 10.8 years, his bone age had advanced to 13 years, but physical exam, LH and testosterone levels remained prepubertal. An ACTH stimulation test disclosed a non-classic congenital adrenal hyperplasia due to 21-hydroxylase deficiency explaining the bone age advancement and, therefore, treatment with cortisone acetate was added. The genetic diagnosis of a homozygous mutation in *GHRHR* (p.Leu144His), a homozygous *CYP21A2* mutation (p.Val282Leu) and *CYP21A1P* pseudogene duplication was established by Sanger sequencing, MLPA and whole-exome sequencing. We report the unusual clinical presentation of a patient born to consanguineous parents with two recessive endocrine diseases: non-classic congenital adrenal hyperplasia modifying the classical GH deficiency phenotype. We used a method of paired read mapping aided by neighbouring mis-matches to overcome the challenges of exome-sequencing in the presence of a pseudogene.

## INTRODUCTION

Isolated growth hormone deficiency (IGHD) is the most common pituitary hormone deficiency; it can be congenital or acquired. Although the most distinctive clinical manifestation is growth failure, there are many other clinical features including a delayed bone age. Amongst the congenital cases, a genetic aetiology can be established in about 10% of patients, with a higher prevalence in familial (34%) compared with sporadic (4%) IGHD. The main genetic causes to date are deleterious mutations in the genes encoding growth hormone (GH), *(GH1)* or the receptor for GHRH (GHRHR) (1).

Congenital adrenal hyperplasia is a genetically heterogeneous disorder, most frequently caused by recessive, loss of function mutations in the 21-hydroxylase enzyme, encoded by *CϒP21A2,* and it can have a wide spectrum of clinical manifestations. The non-classic form can be asymptomatic or associated with signs of postnatal androgen excess: rapid growth, advanced bone age, precocious pubarche, menstrual abnormalities, hirsutism, acne, and/or infertility (2,3). Non-classic CAH due to 21-hydroxylase deficiency is caused mainly by recombination between *CYP21A2* and a nearly identical pseudogene, *CYP21A1P* (4).

Here we report the unusual presentation of a boy with IGHD and advanced bone age due to *GHRHR* and non-classic *CYP21A2* mutations. To our knowledge, the association of these two conditions has not been reported previously. Furthermore, the pitfalls of Sanger and whole-exome sequencing (WES) to reach the genetic diagnosis are discussed.

## CASE REPORT

**Figure 1:**
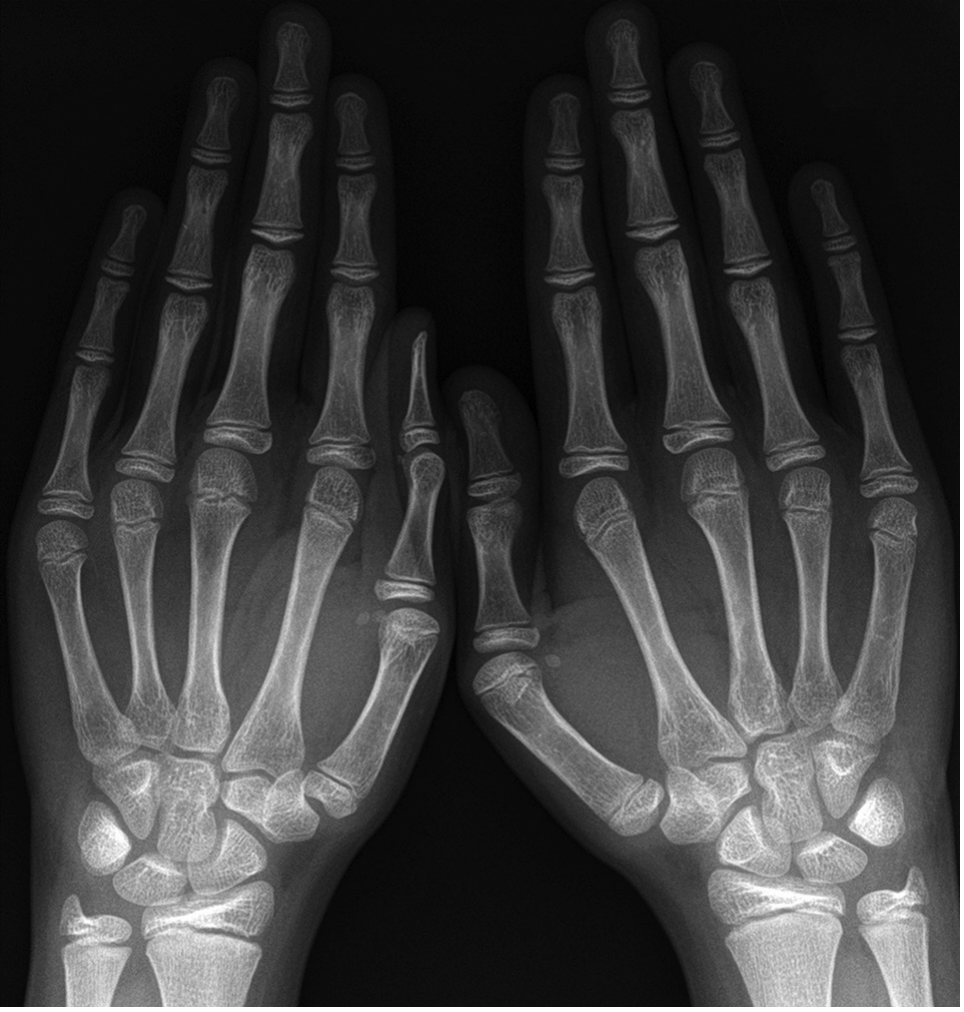
Advanced bone age (13 years) in a patient with chronological age of 10.8 years with isolated growth hormone deficiency.

**Figure 2:**
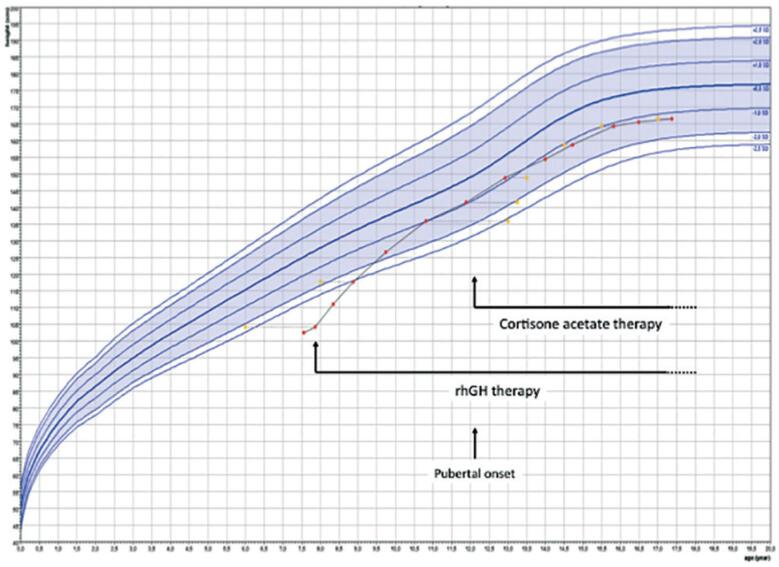
Growth curve. Treatment with recombinant human GH (rhGH) and corstisona acetate are shown. Bone age was determined by the Greulich and Pyle criteria. Growth chart was drawn using Growth analyser 3.5 (Ed. Dutch Growth Foundation, PO Box 23068, 3001 KB, Rotterdam, The Netherlands).

A boy born at term by vaginal delivery (length 50 cm, weight 3,400 g) to second-degree cousins presented at 7.5 years with severe short stature (102.5 cm, SDS-3.7), high-pitched voice, blue sclera and prominent forehead. Genital examination revealed Tanner stage I, absence of pubic hairs, normal penile length (4 cm) and prepubertal testes (length 1.5 cm). Bone age was 6 years according to the Greulich and Pyle method. Basal cortisol (14.8 μg/dL) and FT4 (1.2 ng/dL) were normal. Clonidine stimulation test resulted in a peak GH of 0.6 ng/mL indicating GH deficiency. A combined (insulin, TRH, GnRH) pituitary stimulation tests, performed as part of a research protocol, also showed a peak GH of 0.6 ng/mL and a peak cortisol of 16.1 mcg/dL, initially interpreted as partial ACTH deficiency. Pituitary MRI was normal: anterior lobe 4.6 mm height (normal for age 4.5 ± 0.6 mm) (5), normal pituitary stalk and topic posterior pituitary. The patient was successfully treated with rGH (33 mcg/ kg/day) with a first-year growth velocity of 11.7 cm/ year. Surprisingly, at 10.8 years of age he presented with advanced bone age (13 years), (Figure 1), despite absence of signs of puberty and prepubertal serum LH and testosterone levels. Growth velocity at this point was 8.6 cm/year. An ACTH-stimulation test showed respectively, basal and peak levels, cortisol 6.1 and 18.8 mcg/dL, 17 hydroxyprogesterone 9.4 and 52.0 ng/mL and androstenedione 1.2 and 2.0 ng/mL, indicating non-classic 21-hydroxylase deficiency. The spectrum of clinical manifestations in non-classic CAH is wide and there is no perfect genotype-phenotype correlation (6). We probably found the advanced bone age prior to pubarche because we were checking it periodically due to IGHD. In common clinical settings, pubarche is usually the presenting sign in boys with non-classic CAH. Cortisone acetate (15 mg/m^2^/day) was added to his treatment. At 12.2 years of age pubic hair was Tanner stage 2 and testicular length had increased to 2.5 cm indicating onset of central puberty. At 19.5 years, his adult height was 166.5 cm, below his target height of 178.5 cm, possibly due to his early bone age advancement due to CAH (Figure 2).

## GENETIC TESTING AND DISCUSSION

**Figure 3:**
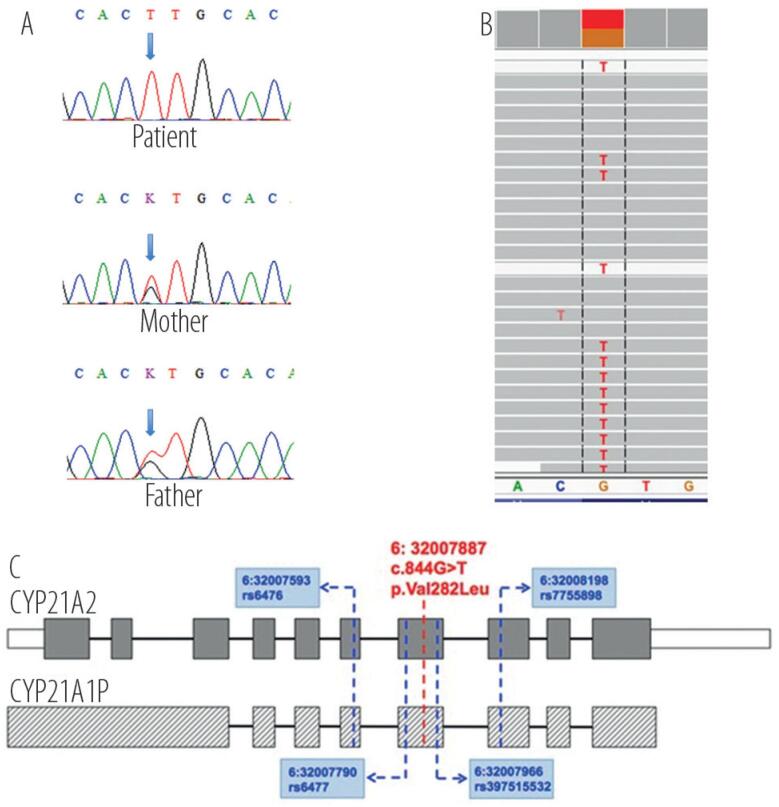
**Panel A:** Sanger sequencing of *CYP21A2* with specific primers showing the mutation c.844G>T (p.Val282Leu) in homozygous state in the patient and in heterozygous state in the parents. **Panel B:** The Integrative Genomics Viewer (IGV) showing Alignment without special bioinformatics treatment suggesting heterozygous state of the *CYP21A2* c.844G>T (p.Val282Leu) mutation. **Panel C:** Diagram of the *CYP21A2* and *CYP21A1P* genes. The p.Val282Leu mutation and genomic position is marked as red. The four mis-matches that were used for pair read mapping between CYP21A2 and CYP21A1P sequences are in blue.

At first, we performed Sanger-sequencing. No mutations in *GH1, GHRH,* or *GHRHR* were found (7). Using specific primers for the active *CϒP21A2* gene (8), a homozygous c.844G>T, p.Val282Leu mutation (previously known as p.Val281Leu) was found (both parents were heterozygous) (Figure 3A). The p.Val282Leu mutation in *CYP21A2* is the most commonly found mutation in patients with non-classic CAH (4) and leads to a mild mutant that retains 2050% of 21-hydroxylase activity (9).

In order to establish the unidentified genetic cause of IGHD, WES was performed. Briefly, we aligned sequence reads to the 1000 Genomes Phase 1 reference mapped to GRCh37 using *BWA v0.5.9* (10) and removed duplicate read pairs using *PICARD v1.74.* We performed realignment, recalibration and variant calling using *GATK v3.3* (11) and applied *GATK VQSR filter* (12) to remove low-quality variants. Variant annotation was retrieved by using ANNOVAR (13) revealing a homozygous c.431C>T, p.Leu144His mutation in *GHRHR*. This mutation is indeed detected in the homozygous state by Sanger sequencing, but it was originally overlooked by the first Sanger sequencing, patient 5 of reference (7). This alteration was not seen when the sequencing data were initially read but after the WES it could be found in the original Sanger sequencing. Here we report the correct *GHRHR* findings for this patient. This mutation has been previously described in unrelated patients with IGHD from Sergipe/Brazil, Pakistan, Spain and the United States, and has been reported to lead to reduced cAMP production after *GHRH* stimulation with normal cell- surface localization of the receptor, suggesting a defect in ligand binding (14). To explore a possible founder effect we analyzed the C/T polymorphisms at positions −261 and −235 of the *GHRHR* promoter, away 7333 and 7359 bp, respectively, from the c.431C>T mutation. Our patient was homozygous C at position −235 and homozygous C at position −261. This haplotype is identical to that of the previously reported patients with p.Leu144His from north-eastern Brazil and Spain but different from the patient from north-eastern United States (15). Therefore, the present patient is not related to the previously reported patient from the United States; however, a common ancestor for both families from Brazil and that of Spain, who share the same haplotype, cannot be excluded. Interestingly, another homozygous *GHRHR* mutation, c.57+1G>A, found in Itabaianinha in the Northeastern Brazilian state of Sergipe, affects the largest kindred of patients with *IGHD* due to *GHRHR* mutations reported to date (16).

As we already had the WES done, we decided to analyse *CϒP21A2* by this method. At first, interpretation of WES indicated the *CYP21A2* p.Val282Leu mutation in heterozygous state (Figure 3B). This was in disagreement with the clinical diagnosis and Sanger sequencing. MLPA, was performed and, revealed *CYP21A1P* and *C4B* duplication. To resolve this gene- pseudogene twist in exome-sequencing, we proposed a method of paired read mapping aided by neighbouring four mis-matches using the exome-sequencing data and manually sorted out the real genotype for the variant of interest at *CYP21A2* (Figure 3C). For the *CYP21A2* mutation, there were 44 paired reads supporting the T allele and 0 supporting the G allele. We concluded that the genotype at *CYP21A2* c.844 position is T/T. For the corresponding *CYP21A1P* mutation, the evidence showed 78 paired reads supporting T and 73 supporting G; thus, it should be G/T at corresponding *CYP21A1P* position. We believe that these difficulties may happen when analyzing mutations in genes with pseudogenes and highly homologous sequences and this methodology can be useful to overcome this limitation.

Ectopic posterior pituitary lobe and an interrupted stalk on MRI are increasingly being used for the diagnosis of GHD. However, it should be noted that all patients with GHD reported to date with mutations in *GH1* and *GHRHR* (as well as in *PROP1)* have had a normal stalk and topic posterior lobe, as the present case did (17,18).

We conclude that in patients with IGHD and advanced bone age clinicians should search for an additional diagnosis. Patients born to consanguineous parents may have more than one genetic disease leading to unusual phenotypes and treatment outcomes. Whole- exome sequencing was able to establish the genetic cause of IGHD but initially presented difficulties in diagnosing the genotype of *CYP21A2/CYP21A1P.* This report reveals the strengths and challenges of each sequencing technology and its applications.
